# The GSK3 Signaling Pathway Is Activated by Cocaine and Is Critical for Cocaine Conditioned Reward in Mice

**DOI:** 10.1371/journal.pone.0088026

**Published:** 2014-02-04

**Authors:** Jonathan S. Miller, Jeffrey L. Barr, Lauren J. Harper, Rachel L. Poole, Thomas J. Gould, Ellen M. Unterwald

**Affiliations:** 1 Department of Pharmacology and the Center for Substance Abuse Research, Temple University School of Medicine, Philadelphia, Pennsylvania, United States of America; 2 Department of Psychology, Temple University, Philadelphia, Pennsylvania, United States of America; Penn State College of Medicine, United States of America

## Abstract

The Akt - GSK3 signaling pathway has been recently implicated in psychostimulant-induced behavioral and cellular effects. Here, the ability of cocaine to regulate the activity of Akt and GSK3 was investigated by measuring the phosphorylation states of the two kinases. The anatomical specificity of the response was determined, as was the contributions of dopamine and NMDA receptors to the actions of cocaine. As GSK3 activity was found to be increased by cocaine, subsequent experiments investigated the importance of GSK3 activation in cocaine conditioned reward. Adult male CD-1 mice were injected with cocaine or saline, and levels of phosphorylated Akt and GSK3α/β were measured 30 minutes later. Acute administration of cocaine significantly decreased the phosphorylation of Akt-Thr308 (pAkt-Thr308) and GSK3β in the caudate putamen and nucleus accumbens core, without altering pAkt-Ser473 and pGSK3α. To investigate the role of dopamine and NMDA receptors in the regulation of Akt and GSK3 by cocaine, specific receptor antagonists were administered prior to cocaine. Blockade of dopamine D2 receptors with eticlopride prevented the reduction of pAkt-Thr308 produced by cocaine, whereas antagonists at dopamine D1, dopamine D2 or glutamatergic NMDA receptors each blocked cocaine-induced reductions in pGSK3β. The potential importance of GSK3 activity in the rewarding actions of cocaine was determined using a cocaine conditioned place preference procedure. Administration of the selective GSK3 inhibitor, SB 216763, prior to cocaine conditioning sessions blocked the development of cocaine place preference. In contrast, SB 216763 did not alter the acquisition of a contextual fear conditioning response, demonstrating that SB 216763 did not globally inhibit contextual learning processes. The results of this study indicate that phosphorylation of GSK3β is reduced, hence GSK3β activity is increased following acute cocaine, an effect that is contingent upon both dopaminergic and glutamatergic receptors. Further, GSK3 activity is required for the development of cocaine conditioned reward.

## Introduction

Cocaine abuse remains one of our society’s major public health problems. Repeated cocaine exposure increases the likelihood of further drug abuse, leading to the development of addiction. Recent neurobiological research has identified neuroadaptations that occur during drug exposure. These adaptations are thought to produce the states of drug reward, dependence, sensitization, withdrawal, and craving, all of which contribute to continued drug-seeking and drug-taking behaviors that form the basis of addiction. As such, the identification of the mechanisms underlying cocaine-induced neuronal plasticity is critically important. One intracellular signaling pathway that has received attention recently for its ability to regulate neuroplasticity and its role in neuropsychiatric disorders is the Akt (protein kinase B) - glycogen synthase kinase-3 (GSK3) pathway. Of direct relevance to drug addiction, Akt and its downstream kinase, GSK3, have been shown to mediate dopaminergic neurotransmission and regulate behaviors including those produced by psychostimulants [1]–[4], as well as modulating behavioral and cellular responses to opiates [5].

Akt is a serine threonine kinase that is a central player in regulation of a number of cellular processes including metabolism, apoptosis, development, and motility [6]. Akt is a downstream component in phospho-inositide 3-kinase (PI3K) signaling which is activated by tyrosine kinases, G-protein coupled receptors, and integrin signaling. Upon signaling through PI3K, Akt translocates from the cytoplasm to the cell membrane where it is activated by phosphorylation of two regulator sites, threonine-308 and serine-473 [7]. Once activated, phosphorylated Akt can translocate to the cytosol or the nucleus. Activated Akt is able to directly phosphorylate and modulate the activity of downstream target proteins which can have wide-ranging cellular effects [8]. One mechanism by which Akt influences cellular processes is through negatively regulating the activity of GSK3 [9].

Originally identified for its regulation of glycogen metabolism [10], GSK3 has been since shown to be critical for a number of cellular processes including synaptic plasticity [11]. GSK3 is also implicated in the pathology of several neuropsychiatric diseases such as schizophrenia and bipolar disorder, as well as being a target for therapeutics used in their treatment [12], [13]. The two isoforms of GSK3 are regulated via phosphorylation of the N-terminal serine-21 (GSK3α) or serine-9 (GSK3β). GSK3 is highly expressed in the mammalian brain including in the frontal cortex, nucleus accumbens, caudate putamen, hippocampus and amygdala [14]. Regulation of GSK3 activity is significant in that it phosphorylates more than 40 substrates including proteins that are important in drug dependence such as the transcription factors CREB and AP-1, the structural proteins dynamin-like protein and neurofilaments, and the signaling proteins PKA regulatory subunit, protein phosphatase 1, and protein phosphatase inhibitor-2 [13], [15].

Previous investigations have demonstrated the importance of the Akt-GSK3 signaling pathway in dopaminergic transmission. Pharmacological inhibition or genetic deletion of the dopamine D2 receptor has been shown to increase phosphorylated Akt-Thr308 and GSK3β in the striatum [16]–[18]. Psychostimulants affecting dopaminergic transmission, such as cocaine and amphetamine, also regulate the activity of Akt and GSK3 [16], [19]–[21]. Inhibition of GSK3 by the non-selective inhibitors, valproate or lithium, or the selective GSK3 inhibitor, SB 216763, blocks hyperactivity induced by cocaine or amphetamine and prevents the development of behavioral sensitization to repeated stimulant administration [2]–[4].

Given the proposed role of the Akt - GSK3 signaling cascade in the striatum in dopaminergic transmission and behaviors [1], the present study measured the impact of acute cocaine administration on the levels of phosphorylated Akt and GSK3 in the caudate putamen, nucleus accumbens and frontal cortex of mice. These brain regions were selected because they are sites at which cocaine inhibits dopamine transporter function, thereby enhancing dopamine neurotransmission [22]–[24]. Acute exposure to cocaine results in elevations of dopamine and glutamate [22]–[25]; hence the roles of dopamine D1 and D2 receptors and the glutamatergic N-methyl-D-aspartate (NMDA) receptor in cocaine-induced regulation of Akt and GSK3 were determined. Cocaine was found to increase GSK3β activity in the nucleus accumbens core and caudate putamen. Therefore, the importance of GSK3 activity in cocaine conditioned reward was investigated using a selective GSK3 inhibitor SB 216763 in a cocaine conditioned place preference assay. Conditioned place preference requires contextual learning to associate the drug (ie, cocaine) with environmental cues. In order to demonstrate the specificity of SB 216763 for cocaine conditioned reward versus potential non-specific effects on contextual learning processes, the effect of SB 216763 on contextual fear conditioning was also determined. Results from these studies demonstrate the ability of cocaine to activate GSK3β and the importance of GSK3 signaling in cocaine reward.

## Materials and Methods

### Animals

Male CD-1 mice (8 weeks old) were obtained from Charles River Laboratories (Wilmington, MA). Mice were housed five per plastic cage without additional enrichment objects in a temperature- and relative humidity-controlled room with a 12-hr light/dark cycle (lights on at 7∶00 a.m.). Animals were housed for seven days prior to testing and were handled and weighed daily. All animals had access to standard laboratory chow and tap water *ad libitum*. All animal testing was conducted in strict accordance with the recommendations of the National Institutes of Health set out in the Guide for the Care and Use of Laboratory Animals and with an approved protocol from Temple University Institutional Animal Care and Use Committee (Animal Welfare Assurance No. A3594-01).

### Drugs

Cocaine hydrochloride was generously supplied by the National Institute on Drug Abuse. SCH-23390, eticlopride hydrochloride and MK-801 were purchased from Sigma-Aldrich (St. Louis, MO). Cocaine, SCH-22390, eticlopride and MK-801 were dissolved in sterile saline (0.9% NaCl) and injected intraperitoneally (i.p.) in a volume of 3 ml/kg of body weight. SB 216763 (Tocris; Ellisville, MO) was dissolved in propylene glycol and brought up to volume in distilled water (70∶30); SB 216763 or vehicle control was injected i.p. in a volume of 10 ml/kg of body weight.

### Drug Administration

To investigate the effect of acute administration of cocaine on Akt and GSK3 activity in the brain, mice were injected with saline (3 ml/kg, i.p.) or cocaine (20 mg/kg, i.p.) and euthanized 30 minutes post-injection. Our preliminary time-course studies, as well as published experiments by ourselves and others [2]; [26], demonstrate that phosphorylation of Akt is reduced 30 minutes after stimulant administration. The involvement of dopamine D1 and D2 receptors and NMDA receptors in cocaine-induced modulation of Akt and GSK3 were assessed using selective receptor antagonists. Mice were injected with the dopamine D1 receptor antagonist SCH-23390 (0.1 mg/kg, i.p.), the dopamine D2 receptor antagonist eticlopride hydrochloride (1 mg/kg, i.p.) or the NMDA receptor antagonist MK-801 (1 mg/kg, i.p.) 30 minutes prior to saline or cocaine (20 mg/kg, i.p.) and euthanized 30 minutes later. Doses of antagonists were selected based on prior studies demonstrating that SCH23390 (0.1 mg/kg), eticlopride (1.0 mg/kg), and MK801 (1.0 mg/kg) inhibit cocaine-induced hyperactivity in mice [27]–[28].

### Immunoblotting (Western Blot)

Thirty minutes following saline or cocaine administration, the frontal cortex, nucleus accumbens (both core and shell regions), and caudate putamen were rapidly dissected from brains on ice according to the mouse atlas of Paxinos and Franklin [29]. Brain regions were dissected from coronal slices AP from bregma as follows: frontal cortex 3.2-1.7; nucleus accumbens 1.7-1.1; caudate putamen 1.7-0.1. Tissues were immediately sonicated in boiling 1% SDS, boiled for 5 minutes, aliquotted and stored at –80°C until assayed. Protein concentrations were determined by a modified Lowry assay [30]. Protein extracts (20 µg) were subjected to SDS-polyacrylamide gel electrophoresis (7.5% Tris-HCl BioRad Ready-gels, Hercules, CA) and transferred for 95 minutes to nitrocellulose membranes. Membranes were subsequently blocked for 1 hour in blocking solution consisting of 5% nonfat dry milk and Tween-TBS and then incubated overnight at 4°C in the following antibodies, pGSK3α/β (1∶2000–1∶5000, Cell Signaling, Beverly, MA), pAkt-Ser473 (1∶1000, Cell Signaling, Beverly, MA), or pAkt-Thr308 (1∶1000, Cell Signaling, Beverly, MA). Following incubation in primary antibodies, membranes were washed in Tween-TBS and incubated in either anti-mouse or anti-rabbit secondary antibody conjugated to horseradish peroxidase (Vector Laboratories, Burlingame, CA) for 1 hour at room temperature. Immunoreactivity was visualized by chemiluminescence following incubation in Supersignal West Pico Chemiluminescent Substrate (Pierce, Rockford, IL) with bands being quantified using the FujiFilm Intelligent Dark Box II, IR LAS-100 Pro V3.1, and Image Gauge V4.22 equipment and software packages. Membranes were stripped and re-probed with either a second primary antibody to quantify levels of total GSK3α/β (1∶10000; Santa Cruz, Santa Cruz, CA) or total Akt (1∶2000; Cell Signaling, Beverly, MA) and β-tubulin (1∶20000; Sigma, St. Louis, MO). Immunoblot data were expressed as a ratio of phosphorylated protein:total protein or total protein:β-tubulin. Data were analyzed by unpaired two-tailed t-test or one-way analysis of variance (ANOVA) followed by planned pair-wise comparisons following a significant ANOVA (GraphPad Prism 5, La Jolla, CA).

### Immunohistochemical Analyses

Immunohistochemical analysis was performed on tissues collected 30 minutes post-injection from mice that were injected with saline or cocaine (20 mg/kg, i.p.). All mice were deeply anesthetized with ketamine hydrochloride (100 mg/kg, i.p.) and perfused transcardially with 4% paraformaldehyde in 0.1 M phosphate buffer (PB; pH 7.4). Immediately after perfusion-fixation, the brains were removed and post-fixed in the same fixative for 1 hr. Forty-micron thick sections were cut through the rostrocaudal extent of the frontal cortex and striatum using a Vibratome (Technical Products, St. Louis, MO) and collected into chilled 0.1 M PB. Sections were then incubated for 30 minutes in PBS-blocking buffer (10% normal goat serum, 0.3% Triton X-100) to inhibit nonspecific antibody binding. Sections were incubated overnight with rabbit anti-phospho-GSK3β-Ser9 (1∶100; Cell Signaling Technology, Beverly, MA) primary antibody diluted in 1% BSA, 0.1% Triton X-100 in PBS, followed by PBS washes and application of Alexa Fluor 555 Conjugate anti-rabbit secondary antibody (Cell Signaling Technology) for 2 hours at room temperature. After three washes in PBS, sections were mounted, air-dried, dehydrated, and cover slipped with cytoseal (Richard-Allan Scientific, Kalamazoo, MI). Immunofluorescence controls consisted of sections in which the primary antibody was omitted or preadsorption of the antibody with its cognate peptide.

#### Bioquantification of immunohistochemistry

To quantify the changes in immunoreactive product, the stained slides were quantified using a Nikon epi-fluorescent microscope with an analog camera and a bioquantification software system (Bioquant TCW 98, Nashville, TN). The assessment and analysis of the data were carried out in a blinded fashion. Camera settings were maintained at a constant level for each acquired image. To ensure consistency in the regions measured in each mouse, 24 (12 bilateral) different microscope fields (0.62 mm^2^) were measured per caudate putamen, 18 (9 bilateral) per frontal cortex, and 12 (6 bilateral) per nucleus accumbens, in 3 sections per animal (at least 200 µm apart). The Videocount Area Array option of the Bioquant software was also utilized for these measurements. Videocount area is defined as the number of pixels in a field that meet a user-defined color threshold of staining multiplied by the area of a pixel at the selected magnification (40X). Color threshold values were selected based on mean levels of immunostaining for pGSK3β. The threshold values were stored in the computer program for consistent auto-measurement of the immunostained slides. Percent area fractions of immunoreaction product were calculated by dividing the videocount area containing pixels at or above the defined background threshold by the videocount area of the total number of pixels in the chosen field, and multiplying by 100. Group means plus standard error of the mean of the percent area fraction were plotted against pretreatment. Differences in immunoreactivity between saline and cocaine groups were analyzed by an un-paired two-tailed t-test with the level of significance set at p<0.05 (GraphPad Prism 5, La Jolla, CA).

### Cocaine Conditioned Place Preference

A randomized unbiased conditioned place preference procedure was used. Conditioned place preference chambers were rectangular in shape (45×20×20 cm) and consisted of two compartments, separated by a removable door. One compartment had a smooth floor with white walls and vertical black stripes, while the other had a sandpaper floor and black walls. On days 1–4, mice were injected with saline (3 ml/kg, i.p.) or cocaine (2.5, 10 or 30 mg/kg, i.p.) and place into alternate sides of the conditioning chamber for 30 minutes. This was repeated for 4 days with mice receiving 2 pairings with saline and 2 pairings with cocaine on alternate sides of the conditioning chamber. On test day (day 5), mice had access to both sides of the conditioning chamber for 30 minutes in a drug-free state and time in each side was recorded. Preference scores were determined by subtracting the amount of time spent in the saline-paired compartment from the cocaine-paired compartment. To investigate the role of GSK3 in the development of cocaine conditioned reward, mice were pretreated with vehicle or SB 216763 (2.5 mg/kg, i.p.) in their home cages 5 minutes prior to cocaine conditioning. This dose of SB 216763 has been shown to reduce the locomotor-stimulating properties of cocaine without altering baseline activity [2].

### Contextual Fear Conditioning

Training and testing of contextual fear conditioning took place in four identical conditioning chambers (17.78×19.05×38.10 cm) housed in sound attenuating boxes (MED Associates, St Albans, VT), as described [31]. The front, back, and top of the chambers were constructed from Plexiglas panels and the side walls were composed of stainless steel. The chamber floors, 18 metal rods spaced 0.6 cm apart, were connected to a shock generator and scrambler and illumination was provided by a 28V bulb located at the top of the left wall. Ventilation fans (69dB), providing background noise and air exchange, were located on the right wall of each sound attenuating box. Stimulus administration was controlled by MED-PC software.

The behavioral procedure was performed as described previously [31]. After habituating for 1 hour, SB 216763 (2.5 mg/kg, i.p.) or vehicle was administered 5 min prior to training in foreground contextual conditioning. Training began with a 148 sec period (baseline) that was followed by a 2 sec unconditioned stimulus (US) (0.62 mA foot-shock). Following the first US was another 148 sec period that was again followed by a 2 sec US (0.62 mA foot-shock). Thirty sec following the 2 sec US, mice were removed from the training chambers and returned to their home cage. The overall training procedure lasted 5.5 min. Contextual testing occurred 24 hours after training. Mice were returned to the original training chambers (context) in a drug-free state for 5 min and freezing behavior was scored. Freezing, defined as the complete absence of movement besides respiration, was sampled for 1 second every 10 seconds during training and testing, and was scored manually. Inter-rater reliability for scoring was 96%.

## Results

### Acute Cocaine Administration Decreased Levels of Phosphorylated Akt-Thr308 in the Caudate Putamen

**Figure 1:**
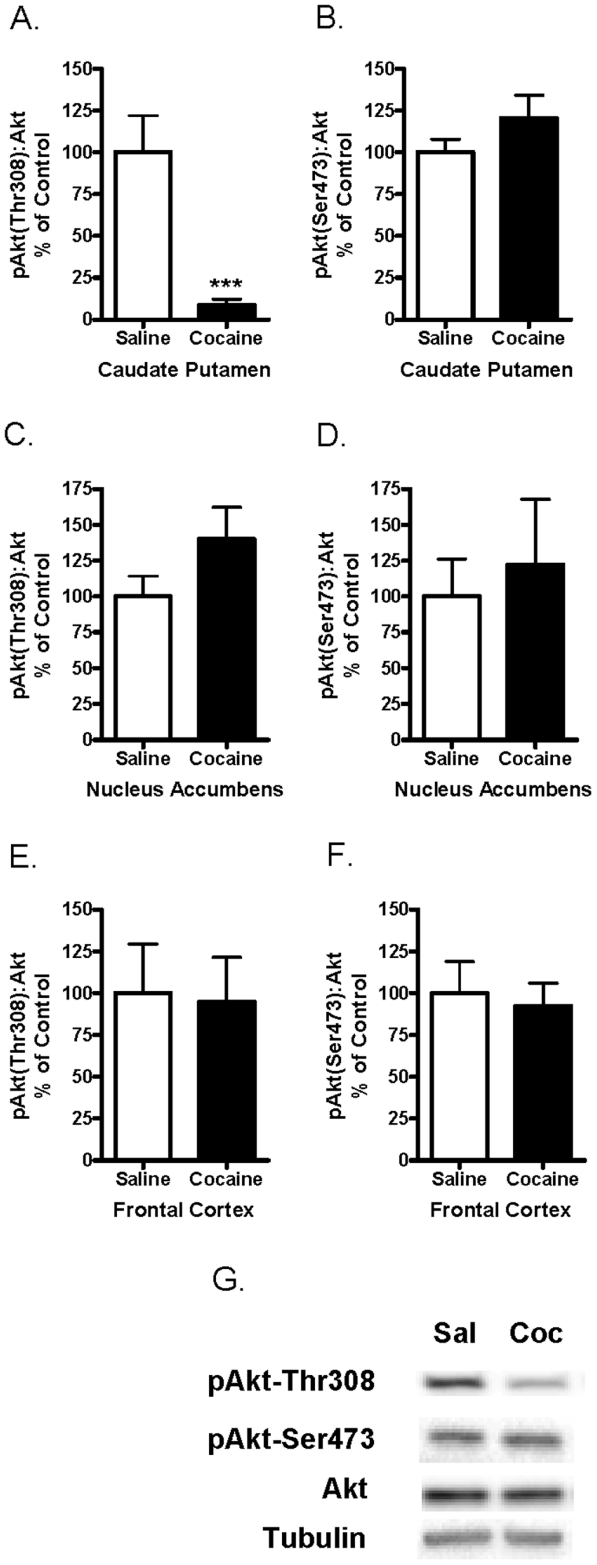
Acute cocaine administration decreased pAkt-Thr308 in the caudate putamen of mice. Levels of pAkt-Thr308 and pAkt-Ser473 were measured by Western blot analysis in mouse caudate putamen, nucleus accumbens and frontal cortex obtained 30 minutes following a single injection of cocaine (20 mg/kg). (1A) Cocaine significantly decreased pAkt-Thr308 (***p<0.001) and (1B) had no effect on pAkt-Ser473 in the caudate putamen. (1C&D) Levels of pAkt-Thr308 and pAkt-Ser473 were not changed in the nucleus accumbens or (1E&F) frontal cortex after cocaine administration. Bars represent the mean ± SEM (N = 7–13/group) and are expressed as a ratio of pAkt:total Akt. Data were analyzed by an unpaired two-tailed Student t-test. (1G) Representative immunoblots of caudate putamen tissue from saline (sal) and cocaine (coc) injected mice. Bands represent pAkt-Thr308, pAkt-Ser473, total Akt, and tubulin (from top to bottom).

Levels of phosphorylated and total Akt were measured by Western blot analysis. Levels of pAkt-Thr308 were significantly lower in the caudate putamen 30 minutes following acute cocaine administration as compared to saline-injected controls (t_23_ = 3.943, p = 0.0006) (Figure 1A). In contrast to the effect in the caudate putamen, cocaine had no effect on pAkt-Thr308 levels in the nucleus accumbens (Figure 1C) (p>0.05) or frontal cortex (Figure 1E) (p>0.05). Acute administration of cocaine did not change the phosphorylation of Akt-Ser473 in any brain region tested (Figures 1B, 1D, 1F) (p>0.05). Levels of total Akt:tubulin were unchanged in all brain regions following cocaine (data not shown). Representative immunoblot images of pAkt-Thr308, pAkt-Ser473, and total Akt from the caudate putamen of a saline- and cocaine-injected mice are shown in Figure 1G. These data demonstrate that acute administration of cocaine reduced the activity of Akt by decreasing the phosphorylation of Akt at its Thr308 residue in the caudate putamen.

### Phosphorylation of GSK3β is Reduced in the Caudate Putamen Following Acute Administration of Cocaine

**Figure 2:**
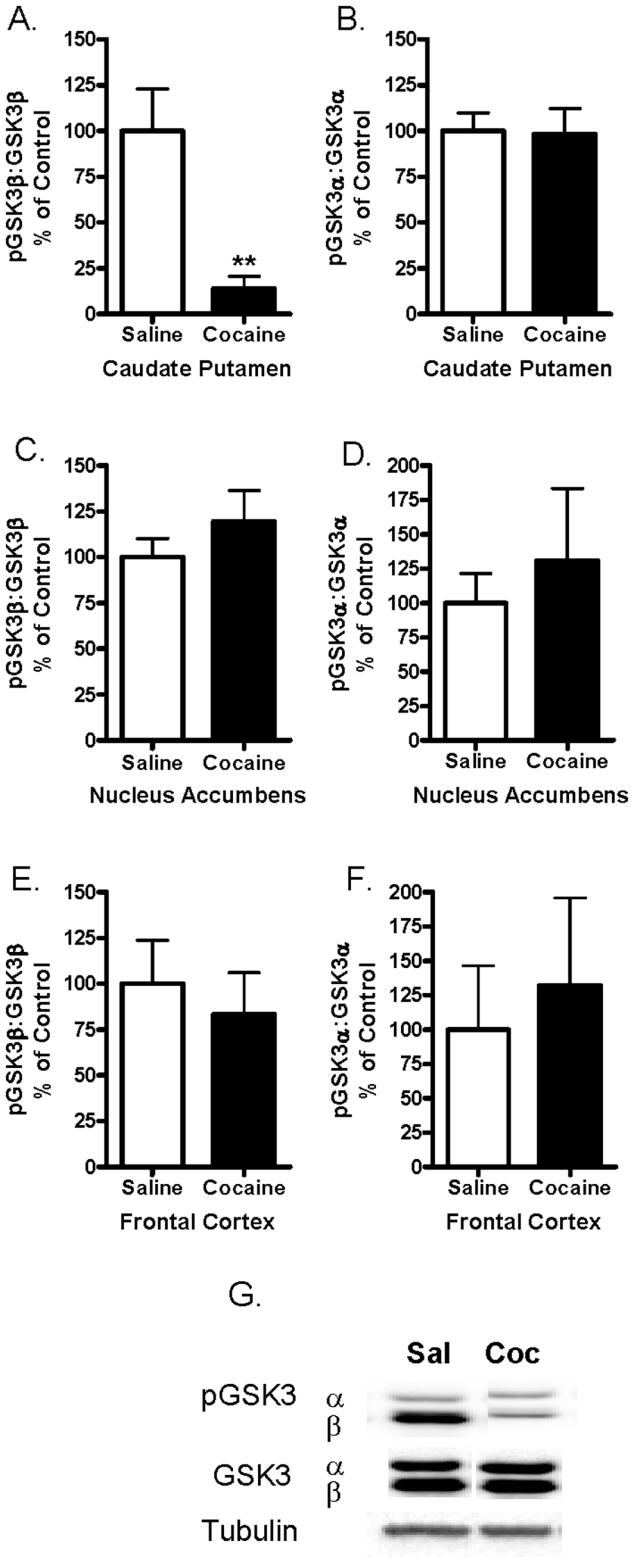
Phosphorylation of GSK3β but not GSK3α was reduced in the caudate putamen following acute administration of cocaine. (2A&B) Cocaine (20 mg/kg) reduced the phosphorylation of GSK3β but not GSK3α in the caudate putamen as compared to saline (**p<0.01). (2C&D) Levels of phosphorylated GSK3α/β were unchanged in the nucleus accumbens (core and shell combined) and (2E&F) frontal cortex 30 minutes following a cocaine injection. Bars represent the mean ± SEM (N = 6–9/group) and are expressed as a ratio of pGSK3α/β:total GSK3α/β. Data were analyzed by unpaired two-tailed Student t-tests. (2G) Representative immunoblots of caudate putamen tissue from saline (sal) and cocaine (coc) treated mice. Bands represent pGSK3α/β, total GSK3α/β and tubulin (from top to bottom).

Protein extracts from the caudate putamen were analyzed for levels of phosphorylated GSK3α, GSK3β and total GSK3α and β by immunoblot. As shown in Figure 2A, mice administered cocaine had significantly less phosphorylated GSK3β in the caudate putamen as compared to saline-injected controls (t_10_ = 3.586, p = 0.005) indicating that GSK3β activity was increased 30 minutes after cocaine administration, as phosphorylation at this site results in decreased kinase activity. In contrast, levels of pGSK3α in the caudate putamen were not changed (p>0.05; Fig. 2B). The abundance of pGSK3α or pGSK3β in the nucleus accumbens (consisting of core and shell) and frontal cortex were not significantly different 30 minutes following cocaine injection as measured by Western blot analysis (p>0.05; Fig. 2C–F). Levels of total GSK3α:tubulin and GSK3β:tubulin were not changed by acute administration of cocaine in the caudate putamen, nucleus accumbens, or frontal cortex (data not shown). Figure 2G shows representative immunoblots of tissue extracts from the caudate putamen probed with antibodies recognizing pGSK3α (51 kDa), pGSK3β (48 kDa), and total GSK3α and β.

### Immunohistochemical Analysis Revealed that Phosphorylation of GSK3β is Reduced in the Caudate Putamen and Core of the Nucleus Accumbens Following Cocaine Administration

**Figure 3:**
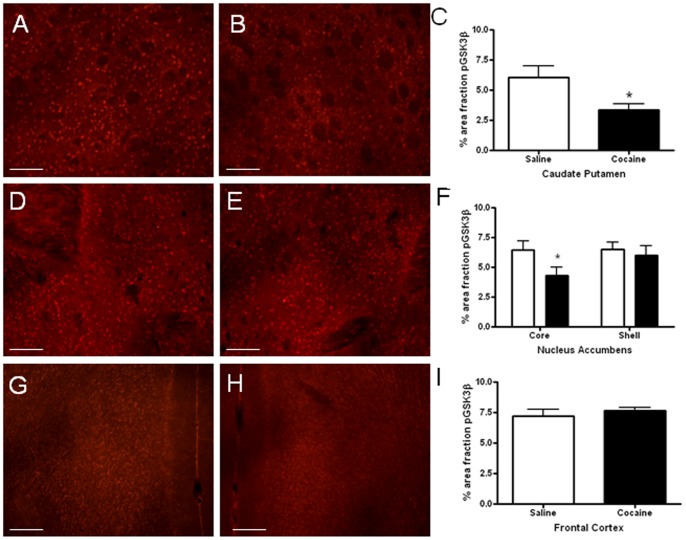
Immunofluorescence labeling of pGSK3β in the mouse brain. Photomicrographs of pGSK3β immunolabeling in the caudate putamen of a mouse injected with saline (3A) or cocaine (3B). Acute administration of cocaine reduced the phosphorylation of GSK3β compared to saline administration in the caudate putamen (*p<0.05; 3C). Photomicrographs of pGSK3β immunolabeling in the nucleus accumbens of mice injected with saline (3D) or cocaine (3E). Levels of phosphorylated GSK3β were significantly lower in the core of the nucleus accumbens (*p<0.05), but not the shell (p>0.05), following cocaine administration (3F). pGSK3β immunolabeling in the frontal cortex of mice injected with saline (3G) or cocaine (3H). No differences were found in pGSK3β immunolabeling in the frontal cortex (3I). Mean ± SEM, (N = 9–10 mice/group). * p<0.05. Scale bar = 50 µm.

Regulation of pGSK3β by cocaine was investigated using a second technique with greater anatomical resolution. Figure 3 shows representative photomicrographs of immunofluorescence labeling for pGSK3β. Immunohistochemical detection of pGSK3β-Ser9 in mouse brain using an antibody selective for pGSK3β produced extensive neuronal labeling. Mice administered cocaine had significantly less pGSK3β-immunoreactivity in the caudate putamen when compared to saline-injected controls (t = 2.470, p = 0.029; Figures 3A, 3B, and 3C). Quantification of pGSK3β-immunoreactivity in the nucleus accumbens showed significantly lower levels of pGSK3β in the core region of the accumbens following cocaine (t = 2.168, p<0.05) but not in the shell (t = 0.643, p>0.05; Figures 3D, 3E, and 3F). In contrast, levels of pGSK3β-immunoreactivity were not significantly different in the frontal cortex 30 minutes following cocaine administration compared to saline-injected controls (p>0.05; Figures 3G, 3H, and 3I). Taken together, these results demonstrate that phosphorylation of GSK3β was significantly reduced in the caudate putamen and the core subregion of the nucleus accumbens 30 minutes after cocaine injection.

### A Dopamine D2 Receptor Antagonist Blocked Cocaine-induced Reduction of pAkt-Thr308

**Figure 4:**
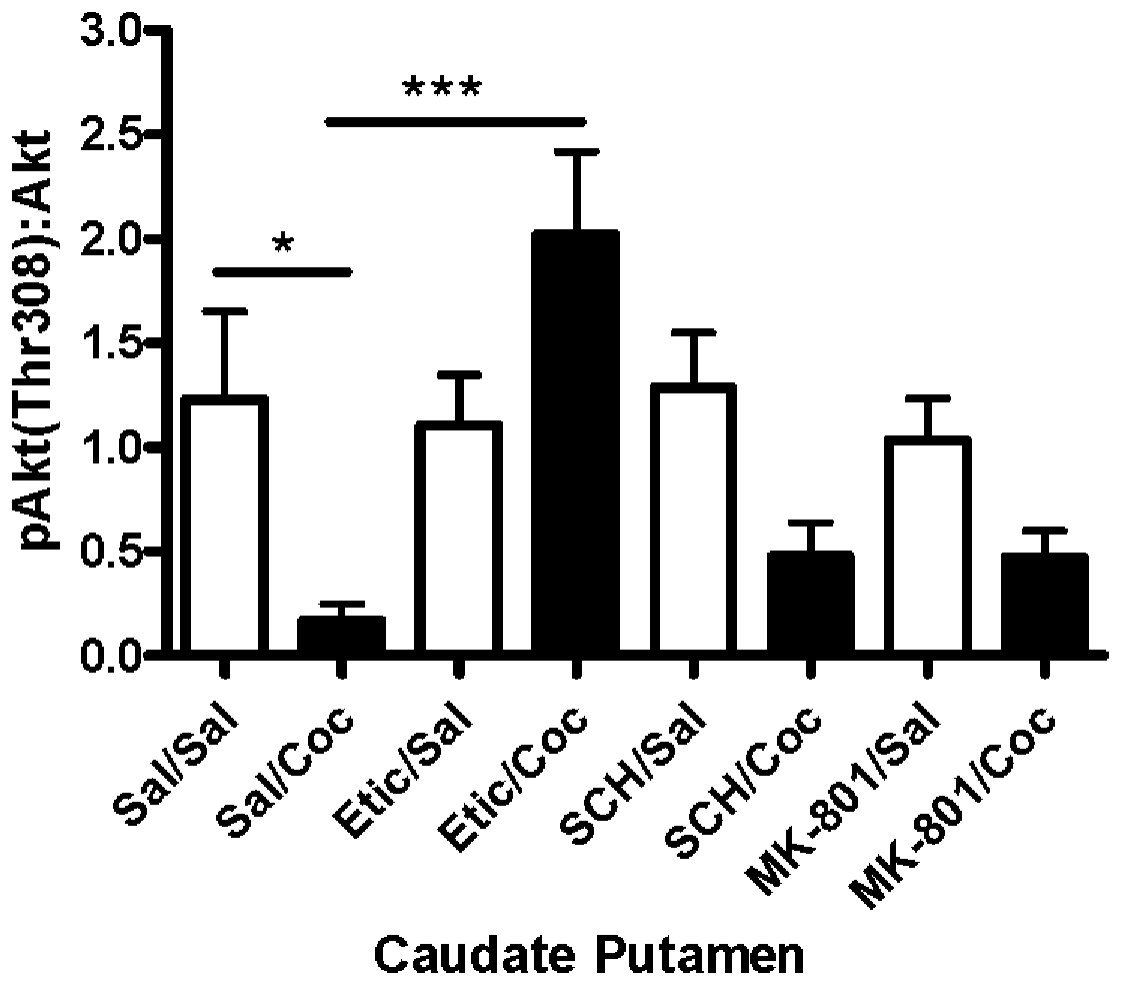
Eticlopride pretreatment attenuated cocaine-induced reduction of Akt-Thr. 308 phosphorylation in the caudate putamen. Acute cocaine administration decreased the levels of pAkt-Thr308 in the caudate putamen (*p<0.05). Administration of eticlopride (1.0 mg/kg) prior to cocaine prevented the cocaine-induced reduction of pAkt-Thr308 (***p<0.001). Administration of the dopamine D1 receptor antagonist SCH-23390 (0.1 mg/kg) or the NMDA receptor antagonist MK-801 (1.0 mg/kg) prior to cocaine did not significantly affect cocaine-induced reductions in pAkt-Thr308. Bars represent the mean ± SEM (N = 5–8/group) and are expressed as a ratio of pAkt:total Akt.

The contributions of D1, D2, and NMDA receptors to the reduction of Akt-Thr308 phosphorylation produced by cocaine in the caudate putamen were assessed by pretreating mice with selective receptor antagonists prior to cocaine administration. Evaluation of significance by ANOVA revealed a significant difference between groups [F(7,47) = 4.48, p = 0.0007; Fig. 4]. Post-hoc analysis showed that cocaine significantly decreased pAkt-Thr308 in the caudate putamen as compared to saline-injected controls (sal/sal vs. sal/coc, p<0.05) in agreement with the data presented in Figure 1A. To investigate the dependence on dopamine D2 receptors, mice were pretreated with the D2 receptor antagonist eticlopride (1 mg/kg, i.p.) 30 minutes prior to saline or cocaine (20 mg/kg, i.p.), and the caudate putamen was collected 30 minutes later. Pretreatment with eticlopride prior to cocaine prevented the cocaine-induced reduction of pAkt-Thr308 (sal/coc vs. etic/coc, p<0.001), indicating the critical role of dopamine D2 receptors in cocaine-induced inactivation of Akt. Eticlopride alone had no significant effect on the level of pAkt-Thr308 in the caudate putamen (sal/sal vs. etic/sal, p>0.05).

Similar studies were performed to determine the role of dopamine D1 receptors in cocaine-induced reductions of pAkt-Thr308. Mice were pretreated with the dopamine D1 receptor antagonist SCH-23390 (0.1 mg/kg, i.p.) 30 minutes prior to cocaine or saline, and the caudate putamen obtained 30 minutes later. Post-hoc analysis showed that administration of SCH-23390 prior to cocaine did not significantly block the cocaine-induced reduction in pAkt-Thr308 level (sal/coc vs. SCH/coc, p>0.05). Administration of SCH-23390 alone had no effect on pAkt-Thr308 (sal/sal vs. SCH/sal, p>0.05; Figure 4).

The involvement of the NMDA receptor in cocaine-induced inactivation of Akt in the caudate putamen was investigated using the NMDA receptor antagonist MK-801. MK-801 pretreatment did not prevent cocaine-induced reductions of Akt-Thr308 phosphorylation (sal/coc vs. MK-801/coc, p>0.05). Administration of MK-801 alone had no effect on the levels of pAkt-Thr308 in the caudate putamen (sal/sal vs. MK-801/sal, p>0.05; Figure 4).

### Pretreatment with a Dopamine D2, D1, or NMDA Receptor Antagonist Attenuated Cocaine-induced Reduction of GSK3β Phosphorylation

**Figure 5:**
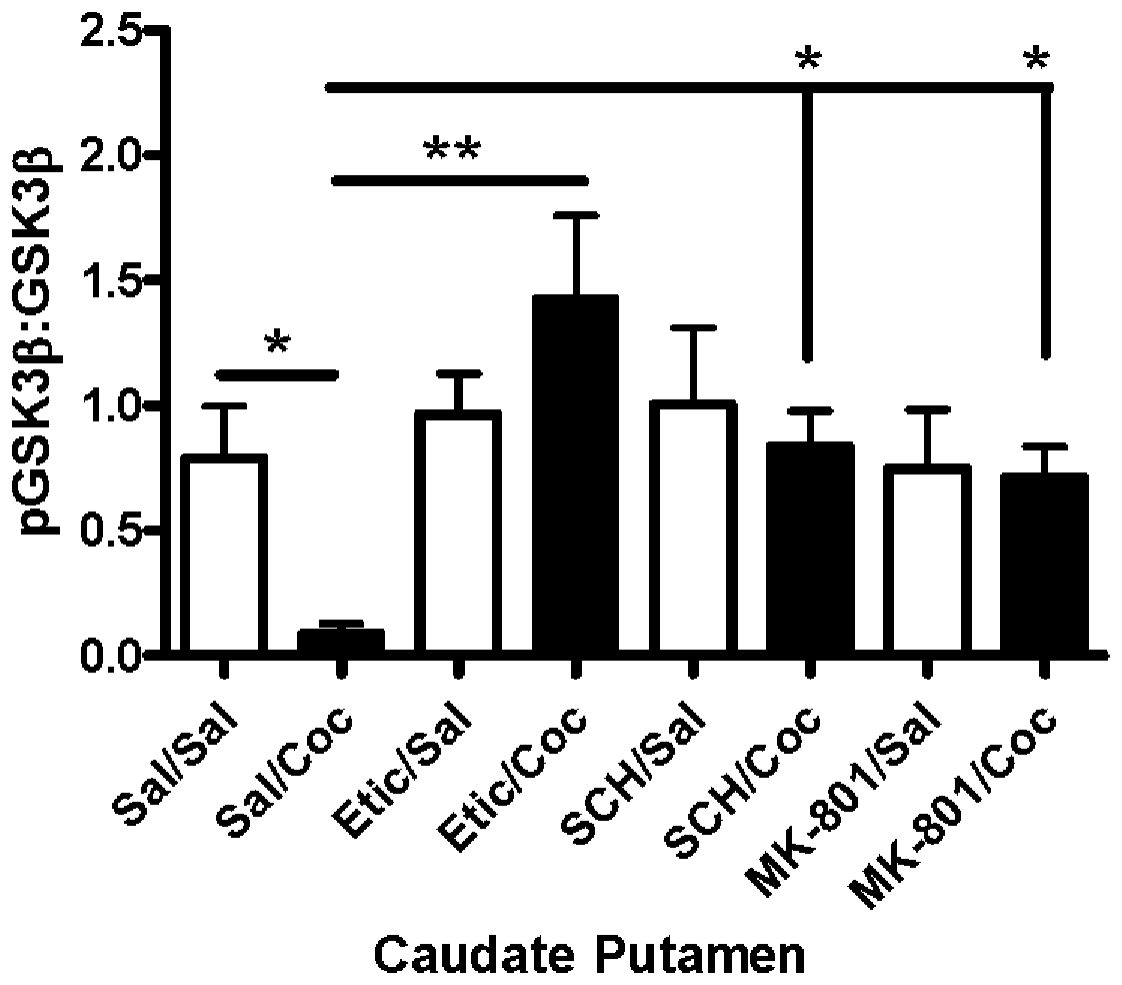
Cocaine-induced reduction of pGSK3β in the caudate putamen was blocked by pretreatment with eticlopride, SCH-23390 and MK-801. Cocaine significantly reduced pGSK3β levels in the caudate putamen 30 minutes after injection (*p<0.05). Pretreatment with eticlopride (1.0 mg/kg) blocked the cocaine-induced decrease in pGSK3β (**p<0.01). Administration of SCH-23390 (0.1 mg/kg) or MK-801 (1.0 mg/kg) prior to cocaine also blocked the reduction of pGSK3β (*p<0.05). Bars represent the mean ± SEM (N = 5–8/group) and are expressed as a ratio of pGSK3α/β:total GSK3α/β.

The roles of D1, D2, and NMDA receptors in the effects of cocaine on GSK3β phosphorylation in the caudate putamen were assessed by pretreating mice with selective receptor antagonists prior to cocaine. ANOVA revealed a significant difference between groups in the levels of pGSK3β [F(7,49) = 2.527, p = 0.026; Figure 5]. Similar to the data presented in Figure 2A, pGSK3β was significantly lower in the caudate putamen of cocaine-injected mice compared with saline controls (sal/sal vs. sal/coc, p<0.05). Pretreatment with D2 receptor antagonist eticlopride prevented the cocaine-induced reduction in pGSK3β (sal/coc vs. etic/coc, p<0.01), while having no effect alone on pGSK3β (sal/sal vs. etic/sal, p>0.05).

To determine if dopamine D1 receptors played a role in cocaine-induced activation of GSK3β, mice were pretreated with the dopamine D1 receptor antagonist SCH-23390 (0.1 mg/kg, i.p.) 30 minutes prior to cocaine or saline. Blockade of dopamine D1 receptors prior to cocaine administration prevented the cocaine-induced reductions in pGSK3β in the caudate putamen (sal/coc vs. SCH/coc, p<0.05). Administration of SCH-23390 alone had no effect on pGSKβ levels (sal/sal vs. SCH/sal, p>0.05).

In addition to dopamine D1 and D2 receptors, the potential involvement of glutamatergic NMDA receptors in cocaine-induced activation of GSK3β in the caudate putamen was investigated. Pretreated with the NMDA receptor antagonist MK-801 (1 mg/kg) 30 minutes prior to cocaine attenuated the decrease in pGSK3β produced by cocaine (sal/coc vs. MK-801/coc, p<0.05). Administration of MK-801 prior to saline did not alter pGSK3β levels in the caudate putamen (sal/sal vs. MK-801/sal, p>0.05). Together, these results indicate that D1, D2, and NMDA receptors are all involved in the reduced phosphorylation of GSK3β in the caudate putamen following acute cocaine administration.

### Inhibition of GSK3 Prevented the Development of Cocaine-induced Place Preference

**Figure 6:**
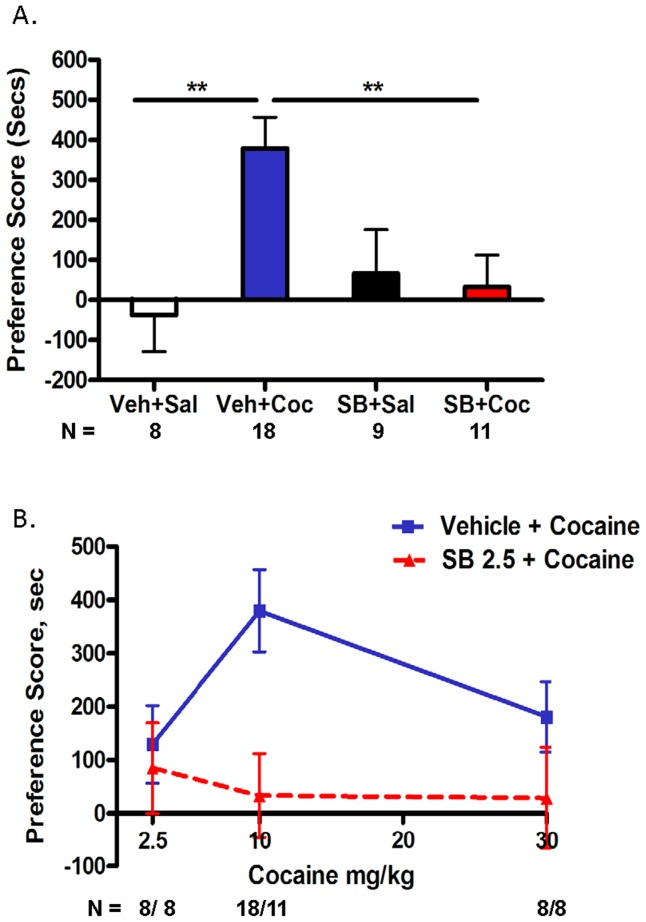
Inhibition of GSK3 prevented the development of cocaine-induced conditioned place preference. Mice were pretreated with vehicle or SB 216763 (2.5 mg/kg, i.p.) followed by cocaine using a 4-day conditioning paradigm. On test day (day 5), preference score (time spent on cocaine-conditioned side minus time spent on saline-conditioned side) was determined. (6A) Mice conditioned with cocaine (10 mg/kg, i.p.) showed a significant place preference toward their cocaine-paired side as compared to saline controls (veh/sal vs veh/coc, **p<0.01). Pretreatment of mice with SB 216763 significantly prevented the development of cocaine-induced conditioned place preference as compared to mice pretreated with vehicle (SB/coc vs veh/coc, **p<0.01). SB 216763 alone had no effect on preference. (6B) There was no significant place preference in mice pretreated with SB 216763 (2.5 mg/kg, i.p.) followed by any dose of cocaine (2.5, 10 or 30 mg/kg). Data were analyzed by two-way ANOVA and Bonferroni post-hoc analysis. All data points are represented as means ± SEM (n = 8–18/group as indicated on figure).

The role of GSK3 activity in cocaine-conditioned reward was evaluated by pretreating the mice with the selective GSK3 inhibitor SB 216763 (2.5 mg/kg) prior to administration of cocaine (10 mg/kg) in the conditioned place preference procedure. Two-way ANOVA of the place preference data shown in Figure 6A revealed significant interaction and treatment effects (Interaction: F(1,42) = 6.829, p = 0.0124; Pretreatment: F(1,42) = 1.987, p = 0.1661; Treatment: F(1,42) = 4.977, p = 0.0311). Bonferroni post-hoc analysis indicated that mice conditioned with 10 mg/kg cocaine demonstrated a significant preference toward their cocaine-paired side as compared to saline controls (**p<0.01; veh/sal vs. veh/coc). Mice pretreated with SB 216763 five minutes prior to cocaine conditioning showed no preference toward their cocaine-paired side as compared to animals pretreated with vehicle prior to cocaine (**p<0.01; SB/coc vs. veh/coc). SB 216763 alone had no effect on place preference (p>0.05; veh/sal vs. SB/sal). The effects of SB 216763 on two other doses of cocaine were also tested (Figure 6B). There was no significant place preference in mice pretreated with SB 216763 (2.5 mg/kg) followed by any dose of cocaine tested (2.5, 10 or 30 mg/kg). Thus, selective inhibition of GSK3 during cocaine conditioning prevented the development of cocaine-induced place preference.

### Inhibition of GSK3 did not Affect the Development of Contextual Fear Conditioning

**Figure 7:**
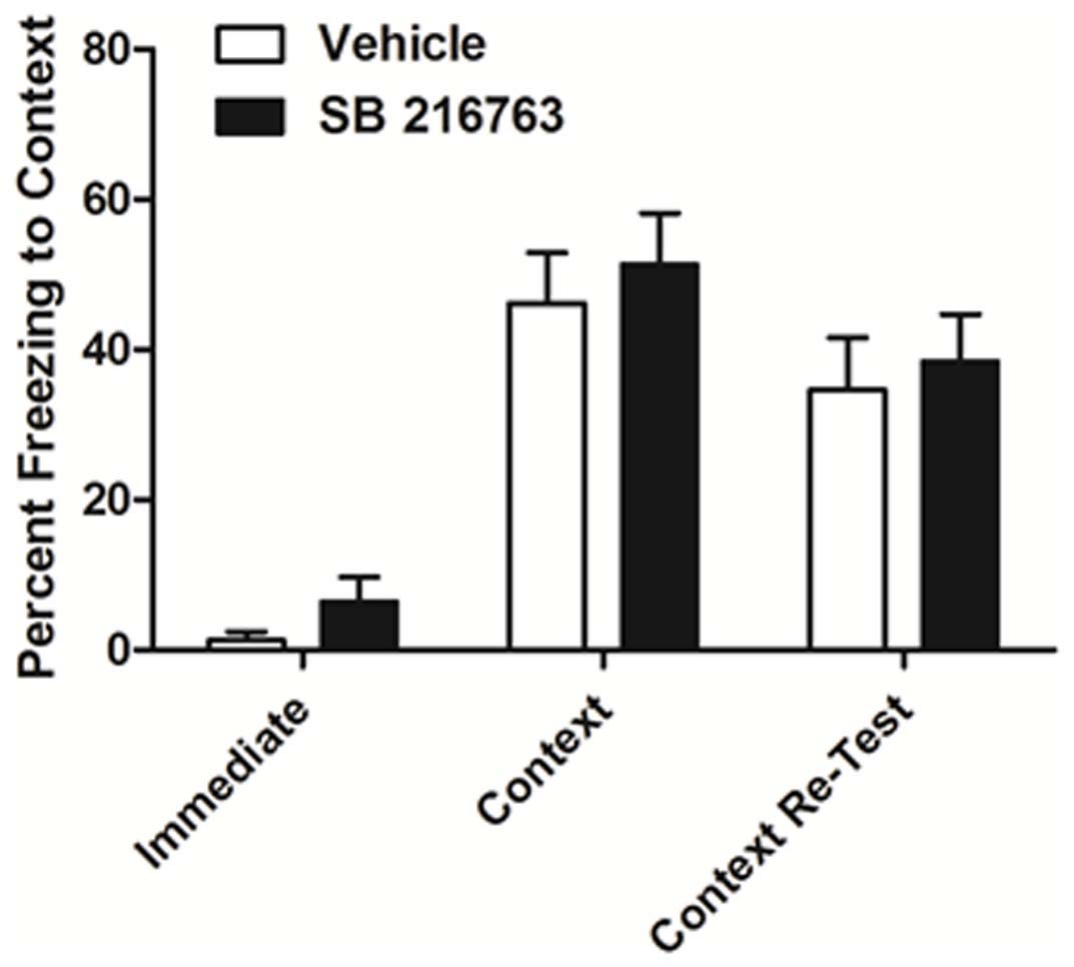
Inhibition of GSK3 did not alter the development of contextual fear conditioning. Mice were injected with vehicle or SB 216763 5 minutes prior contextual fear conditioning. There was no difference in freezing to context between groups when retested 24 hours later. Data points are represented as means ± SEM (n = 13/group).

The successful establishment of a conditioned place preference requires intact contextual learning and memory. In order to rule out the possibility that SB 216763 interfered with learning processes rather than cocaine reward itself, the effect of SB 216763 on the acquisition of contextual fear conditioning was tested. Administration of SB216763 (2.5 mg/kg) prior to the training session did not alter freezing to context when mice were re-exposed to the context 24 hours later (Figure 7; vehicle vs SB 216763, p>0.05). These data indicate that SB 216763 did not interfere with contextual learning required for the development of a fear conditioned response.

## Discussion

The findings of this study demonstrate that acute cocaine administration reduced the phosphorylation of Akt at the Thr308 regulatory site in the caudate putamen which corresponds to a decrease in Akt activity. The phosphorylation of GSK3β was also reduced in the caudate putamen following cocaine, indicating an increase in GSK3β activity as phosphorylation of Ser21 of GSK3β inhibits kinase activity. Increases in GSK3β activity also occurred in the core region of the nucleus accumbens. The regulatory effect of cocaine on GSK3β was contingent upon activation of dopamine D1, D2 and NMDA receptors, whereas its effect on Akt was dependent on D2 receptors only. Dopaminergic and glutamatergic systems are critical to the behavioral and cellular effects of cocaine. Cocaine indirectly activates dopamine receptors, an effect that is achieved by cocaine binding to and inhibiting the dopamine transporter, thereby increasing extracellular dopamine levels [22], [32], [33]. Likewise, extracellular glutamate is increased after cocaine, which is thought to be dependent on dopamine D1 receptors [25], [34]. Activation of dopamine and glutamate receptors following cocaine alters a number of intracellular signaling proteins and subsequently has effects on behavior and gene expression.

Multiple lines of investigation have provided support for the signaling of dopamine receptors through Akt, particularly for D2 receptors. D2 receptors can signal through G-protein independent mechanisms involving a β-arrestin complex that scaffolds to Akt [1]. D2 receptor agonists can regulate the phosphorylation of Akt and GSK3 [35]. Mice lacking dopamine D2 receptors display an increase in pAkt-Thr308 and pGSK3β in the striatum [18], similar to the effects of D2 receptor antagonists [36]. The data presented here are consistent with previous studies supporting the involvement of dopamine D2 receptors in the regulation of the Akt - GSK3 signaling pathway. Pretreatment with the dopamine D2 receptor antagonist eticlopride prevented cocaine-induced reductions in the phosphorylation of Akt-Thr308 and GSK3β, demonstrating that activation of dopamine D2 receptors is essential to cocaine-induced regulation of Akt and GSK3 activity. In a prior report, eticlopride was shown to block amphetamine-induced reductions in pAkt-Thr308 in rat striatum when measured two hours after the amphetamine injection, while having no effect on amphetamine-induced increases in pAkt-Thr308 at 15 minutes following amphetamine injection [16].

Perhaps surprisingly, the present data indicate that blockade of dopamine D1 receptors attenuated cocaine-induced activation of GSK3β. GSK3 has been shown to be involved in dopamine D1 receptor-mediated hyperactivity, as selective inhibition of GSK3 attenuates hyperactivity produced by administration of the D1 receptor agonist SKF-82958, albeit the attenuation is not complete [37]. Previous investigations focusing on the relationship between dopamine D1 receptors and the Akt - GSK3 signaling cascade report contradictory findings. Administration of the D1 receptor antagonist SCH-23390 to mice lacking the dopamine transporter has no effect on pAkt-Thr308 or pGSK3α/β levels in the striatum [19]. D1 receptor knockout mice however show lower levels of pAkt-Ser473 in the striatum than wild-type controls [18] and D1 receptor agonists increase pAkt-Thr308 in primary striatal neuronal cultures [38].

In the present study, pretreatment with the dopamine D1 receptor antagonist SCH-23390 prior to cocaine prevented cocaine-induced reductions in pGSK3β but not pAkt-Thr308 in the caudate putamen. This indicates that the dopamine D1 receptor can affect the phosphorylation of GSK3 in the absence of regulating Akt activity. Therefore, it is tempting to speculate that another kinase may be involved in regulating the phosphorylation of GSK3β in response to dopamine D1 receptor activation. It may be that GSK3 regulation following cocaine is partly contingent upon calcium-dependent signal transduction. Dopamine D1 receptors influence calcium-dependent signaling by coupling to Gq proteins [39] and releasing calcium from intracellular stores [40]. Activation of Gq increases the activity of GSK3β and is not dependent on Akt [41]. Alternatively, it has been shown that amphetamine-induced regulation of pGSK3β in mouse striatum is dependent on DARPP-32 signaling which is downstream from D1 receptor activation [42]. Thus, cocaine may be regulating GSK3 activity at least partially through stimulation of D1 receptor and subsequent activation of Gq or DARPP-32 signaling.

In addition to dopamine receptors, other receptors can modulate the activity of Akt and GSK3β suggesting that the acute effect of cocaine on Akt and GSK3 may involve non-dopaminergic receptors. Since cocaine increases extracellular glutamate in the caudate putamen [25] and since the glutamatergic NMDA receptor antagonist MK801 blocks cocaine conditioned place preference [43], the ability of MK-801 to block the regulation of Akt and GSK3 following cocaine was investigated. Blockade of NMDA receptors prevented the cocaine-induced decrease in pGSK3β in the caudate putamen. Our results are consistent with a previous investigation demonstrating that stimulation of NMDA receptors can activate GSK3 (reduces pGSK3) via protein phosphatase-1 in the adult mouse brain [44]. Pretreatment with the NMDA receptor antagonist prior to cocaine did not affect the cocaine-induced inhibition of Akt, suggesting that regulation of Akt following cocaine is not contingent upon activation of the NMDA receptor and further supporting that GSK3β regulation can be independent from Akt.

The Akt - GSK3 signaling cascade plays an important role in the behavioral effects of psychostimulants. Our previous studies as well as results from others have demonstrated that GSK3 is critical to the acute and sensitized hyper-locomotor responses to psychostimulants; selective and non-selective GSK3 inhibitors can block cocaine- and amphetamine-induced motor activity and sensitization [2]–[4]. Likewise, heterozygote GSK3β mice with reduced levels of GSK3β display an attenuated hyper-locomotor response to amphetamine as compared with wild-type mice [19]. The data presented herein are the first to show that selective inhibition of GSK3 prevented the development of cocaine conditioned place preference, a measure of the rewarding properties of cocaine. It is well established that both dopaminergic and glutamatergic transmission contribute to the development of cocaine-conditioned reward. Given the role of GSK3 in dopamine and glutamate receptor-mediated signal transduction and our findings that cocaine-induced activation of GSK3β can be inhibited by both dopamine receptor and NMDA receptor blockade, it may be that both dopamine and glutamate receptor signaling through GSK3 are important for cocaine-conditioned reward.

The disruption of the development of cocaine place preference was specific in this study, as the same dose of SB216763 administered 5 minutes prior to contextual fear conditioning failed to attenuate the development of a contextual fear response. The finding that inhibition of GSK3 with SB216763 prior to cocaine conditioning sessions prevented the development of a cocaine place preference could be due to attenuation of the rewarding properties of cocaine at the time of the conditioning sessions or, alternatively, to general interference with conditioned learning. Fear conditioning was chosen as a control for investigating non-specific effects of SB 216763 on conditioning. Fear conditioning involves the hippocampus and amygdala [45]–[47] and is sensitive to alterations in striatal function [48]–[50]. Thus, if changes in GSK3 signaling were producing non-specific effects on conditioned learning through actions in these brain regions, altered contextual and/or cued fear conditioning would be expected to be seen. This was not the case, lending further support for the conclusion the SB216763 attenuated cocaine conditioned place preference by interfering with the positive rewarding effects of cocaine.

Dopamine D1 receptor stimulation is important for the development of cocaine conditioned place preference as antagonism of the D1 receptor during cocaine conditioning prevents the acquisition of cocaine place preference [51]. The dopamine D1 receptor also functions as a primary reward in cocaine-naïve animals as evidenced by the findings that D1 receptor agonists themselves can induce place preference [52] and are self-administered [53]. In contrast, pharmacological inhibition of the dopamine D2 receptor does not affect the induction of cocaine place preference [51] and administration of the dopamine D2 receptor agonist quinpirole fails to produce place preference in cocaine-naïve animals [52]. Glutamatergic NMDA receptors are also involved in the development of cocaine-induced place preference as pharmacological and genetic inhibition of NMDA receptors prevents the development of cocaine-induced place preference [43], [54], [55]. It is possible that SB 216763, by inhibiting GSK3, blocked the signaling of both dopamine and NMDA receptors which are necessary for producing cocaine conditioned reward.

In the present study, acute administration of cocaine reduced the phosphorylation of Akt-Thr308 and GSK3β in the caudate putamen. These results are consistent with those indicating that acute amphetamine also reduces pAkt-Thr308 and pGSK3β in the striatum of mice [19]. Analysis of brain sections stained with the anti-pGSK3β antibody showed a significant reduction in pGSK3β-immunoreactivity in the core region of the nucleus accumbens, in addition to the caudate putamen of cocaine-injected mice. The neural substrate of cocaine reward is largely attributed to the nucleus accumbens [56]. In addition, the dorsal striatum (ie, caudate putamen) is strongly implicated in reward mechanisms based on habit learning theories of addiction [56]–[57]. In the present study, SB 216763 was administered systemically and thus inhibited GSK3 in all brain regions. The site of action of SB 216763 in attenuating cocaine place preference has yet to be determined, but may involve the nucleus accumbens core or dorsal striatum where GSK3β was shown to be activated by cocaine.

In summary, the data presented herein demonstrate that the phosphorylation of Akt and GSK3β are reduced by acute exposure to cocaine, leading to heightened activity of GSK3β in the caudate putamen and nucleus accumbens core. Activation of GSK3β by cocaine was contingent upon dopamine D1, D2 and NMDA receptor activation, whereas reduced phosphorylation of Akt was dependent upon dopamine D2 receptors only. These data point to the engagement of multiple pathways in the heightened activity of GSK3β following cocaine. The development of cocaine conditioned place preference was found to be contingent on activation of GSK3. Taken together, the results highlight the importance of the GSK3 signaling pathway in the rewarding effects of cocaine and suggest that regulation of the Akt - GSK3 pathway may be important in cocaine-induced neuroplasticity.
